# RTA 408, A Novel Synthetic Triterpenoid with Broad Anticancer and Anti-Inflammatory Activity

**DOI:** 10.1371/journal.pone.0122942

**Published:** 2015-04-21

**Authors:** Brandon L. Probst, Isaac Trevino, Lyndsey McCauley, Ron Bumeister, Irina Dulubova, W. Christian Wigley, Deborah A. Ferguson

**Affiliations:** Reata Pharmaceuticals Inc., Irving, Texas, United States of America

## Abstract

Semi-synthetic triterpenoids are antioxidant inflammation modulator (AIM) compounds that inhibit tumor cell growth and metastasis. Compounds in the AIM class bind to Keap1 and attenuate Nrf2 degradation. In the nucleus, Nrf2 increases antioxidant gene expression and reduces pro-inflammatory gene expression. By increasing Nrf2 activity, AIMs reduce reactive oxygen species and inflammation in the tumor microenvironment, which reverses tumor-mediated immune evasion and inhibits tumor growth and metastasis. AIMs also directly inhibit tumor cell growth by modulating oncogenic signaling pathways, such as IKKβ/NF-κB. Here, we characterized the in vitro antioxidant, anti-inflammatory, and anticancer activities of RTA 408, a novel AIM that is currently being evaluated in patients with advanced malignancies. At low concentrations (≤ 25 nM), RTA 408 activated Nrf2 and suppressed nitric oxide and pro-inflammatory cytokine levels in interferon-γ-stimulated RAW 264.7 macrophage cells. At higher concentrations, RTA 408 inhibited tumor cell growth (GI_50_ = 260 ± 74 nM) and increased caspase activity in tumor cell lines, but not in normal primary human cells. Consistent with the direct effect of AIMs on IKKβ, RTA 408 inhibited NF-κB signaling and decreased cyclin D1 levels at the same concentrations that inhibited cell growth and induced apoptosis. RTA 408 also increased CDKN1A (p21) levels and JNK phosphorylation. The in vitro activity profile of RTA 408 is similar to that of bardoxolone methyl, which was well-tolerated by patients at doses that demonstrated target engagement. Taken together, these data support clinical evaluation of RTA 408 for cancer treatment.

## Introduction

Antioxidant inflammation modulators (AIMs) include synthetic derivatives of oleanolic acid, a triterpenoid found in medicinal plants [1]. As a class, the AIMs exhibit potent anti-inflammatory and anti-carcinogenic activity due to their ability to activate the transcription factor nuclear factor, erythroid 2-like 2 (NFE2L2 or Nrf2) and inhibit the activity of nuclear factor kappa B (NF-κB). Oleanolic acid itself is a weak Nrf2 activator [2]; however, key alterations to the triterpenoid scaffold improved potency by more than 6 orders of magnitude [3]. The adaptor protein Kelch-like ECH-associated protein 1 (Keap1) targets Nrf2 for Cul3-Rbx1-mediated ubiquitination and constitutive proteasomal degradation, thereby maintaining low basal levels of Nrf2 [4]. AIMs increase Nrf2 levels by binding to Keap1 and blocking its ability to promote Nrf2 degradation [3,5]. As a result, newly synthesized Nrf2 accumulates in the nucleus where it increases the expression of antioxidant genes and decreases the expression of pro-inflammatory genes [6,7].

The Keap1/Nrf2 pathway is the primary target of AIMs at lower concentrations that reduce oxidative stress and inflammation [8]. However, multiple oncogenic signaling pathways are modulated at higher concentrations of AIMs that inhibit tumor cell growth [9,10]. For example, AIMs directly inhibit NF-κB signaling by binding to inhibitor of kappa light polypeptide gene enhancer in B-cells, kinase beta (IKBKB or IKKβ) [11–13]. Other proteins that are dysregulated in cancer are also affected by AIMs, including: JNK [14]; JAK1 and STAT3 [15,16]; Her2 (ERBB2) [17]; death receptor 5 (TNFRSF10B) [18]; and cFLIP (CFLAR) [19]. By modulating the activity of these proteins in the tumor and reducing oxidative stress and inflammation in the tumor microenvironment, AIMs inhibit several pro-tumor processes, including cell proliferation, angiogenesis, inflammation, metastasis, tumor-mediated immune evasion, and suppression of apoptosis [20–26].

Bardoxolone methyl (RTA 402, CDDO-Me) is an AIM with potent anticancer activity in vitro and in animal models [9]. Doses of bardoxolone methyl that increased expression of the classic Nrf2 target gene *NQO1* and decreased tumor levels of NF-κB and cyclin D1 were well-tolerated by patients with advanced malignancies in a phase 1 trial (ClinicalTrials.gov ID: NCT00529438) [27]. In this trial, one patient with mantle cell lymphoma exhibited a complete response and another with anaplastic thyroid carcinoma exhibited a partial response that lasted 18 months. These promising preliminary findings support continued development of AIMs as a novel approach to cancer treatment.

A novel compound in the AIM class, RTA 408, is currently under investigation in a phase 1 clinical trial in patients with metastatic non-small cell lung cancer or melanoma (clinicaltrials.gov ID: NCT02029729). The anti-inflammatory activity of RTA 408 was recently demonstrated in a model of radiation-induced dermatitis [28,29], but its anticancer activity has not previously been reported. In the present study, we evaluated the effect of RTA 408 on tumor cell growth, apoptosis, and oncogenic signaling pathways. We first evaluated the potency of RTA 408 as an activator of Nrf2 and an inhibitor of inflammation in the RAW 264.7 mouse macrophage cell line. We next assessed the effect of RTA 408 treatment on the growth and survival of human tumor cell lines of different origin. Finally, we evaluated the effect of RTA 408 on markers of cellular proliferation and apoptosis.

## Materials and Methods

### Materials

RTA 408 and bardoxolone methyl were synthesized by Reata Pharmaceuticals, Inc. (Irving, TX). Unless noted, all other chemicals were purchased from Sigma-Aldrich. Wild-type and *Keap1* ^-/-^ murine embryonic fibroblasts (MEFs) were from Dr. Masayuki Yamamoto (Tohoku University, Japan) [30,31]. A549/NF-κB-Luc and HeLa/NF-κB-Luc stable cell lines were from Panomics (Fremont, CA). Normal human dermal fibroblasts (NHDF), normal human lung fibroblasts (NHLF), and normal human mesangial cells (NHMC) were purchased from Lonza. All other cell lines were from the American Type Culture Collection (ATCC).

### Cell culture and treatment

MEFs, PANC-1, A549, A375, A549/NF-κB-Luc and HeLa/NF-κB-Luc cells were cultured in Gibco high glucose DMEM (Life Technologies) with 10% FBS. G-361 cells were cultured in McCoy’s 5A medium (Life Technologies) with 10% FBS. All other cell lines were cultured in RPMI 1640 medium with 10% FBS. Culture media for all cell lines was supplemented with 1% penicillin/streptomycin. Media for A549/NF-κB-Luc and HeLa/NF-κB-Luc cells also contained 0.1 mg/mL hygromycin-B. NHDF and NHLF cells were cultured in fibroblast basal medium (FBM) supplemented with 0.1% insulin, 0.1% rhFGF-B, 0.1% GA-1000, and 2% fetal bovine serum (Lonza). NHMCs were cultured in mesangial cell basal growth medium (MsBM) supplemented with 5% fetal bovine serum and 0.1% GA-1000 (Lonza). Cells were cultured in a humidified atmosphere at 37°C with 5% CO_2_. RTA 408 and bardoxolone methyl were dissolved in DMSO (vehicle). The final amount of DMSO in the media was ≤ 0.1% and was equivalent in drug- and vehicle-treated samples.

### Nitric Oxide Assay (Griess Reaction)

RAW 264.7 cells were seeded in 96-well plates at 3 x 10^4^ cells per well in RPMI 1640 medium with 0.5% FBS. The following day, cells were treated with RTA 408 or bardoxolone methyl. Two hours later, 20 ng/mL IFNγ (RD Systems) was added to each well and cells were incubated for an additional 24 hours. Nitrite (NO_2_^-^) levels were measured in media as a surrogate for nitric oxide using the Griess Reagent System (Promega). Cell viability was assessed using Cell Proliferation Reagent WST-1 (Roche Applied Science).

### Messenger RNA quantification

Total RNA was isolated from cells with the RNeasy Mini Kit (Qiagen) and reverse transcribed using iSCRIPT (Bio-Rad). Real-time PCR was performed using iQ SYBR Green Supermix in a CFX96 Real-Time PCR Detection System (Bio-Rad). PCR reactions were performed using validated primers (S1 Table). Ribosomal protein S9 (RPS9) and ribosomal protein L19 (Rpl19) were used as reference genes for human and mouse samples, respectively. The relative abundance of each target gene was determined using the 2^-ΔΔCT^ method [32].

### Western blots

For RAW 264.7 cells, cells were washed with PBS and lysed in Tricine Sample Buffer (Bio-Rad) containing 2% BME. Whole cell lysates were heated to 100°C for 10 minutes and stored at -20°C. For tumor cell lines, cells were scraped into 1 mL media, centrifuged at 2000 x *g*, and washed with PBS. Pellets were resuspended in lysis buffer [20 mM HEPES (pH 7.4), 1.5 mM MgCl_2_, 1 mM DTT, 10 mM KCl, 1 mM EGTA, 1 mM EDTA, 1% Triton X-100, Complete Protease Inhibitor Cocktail (Roche Applied Science), and Phosphatase Inhibitor Cocktail 3]. Protein concentration was determined using DC Protein Assay (Bio-Rad). Proteins (20 to 40 μg) were resolved by SDS-PAGE, and transferred to nitrocellulose membranes. Membranes were incubated with primary antibodies overnight at 4°C. Primary antibody information can be found in S2 Table. AffiniPure HRP-conjugated goat anti-rabbit, donkey anti-goat, and goat anti-mouse IgG secondary antibodies were from Jackson ImmunoResearch.

### Protein quantification in media

RAW 264.7 cells were plated at 1.5 x 10^5^ cells per well in 24-well plates and treated with RTA 408 the following day. Two hours later, 20 ng/mL IFNγ (RD Systems) was added to each well and cells were incubated for an additional 24 hours. Following this incubation, Ccl2 (Mcp1) and Ccl5 (Rantes) protein concentrations in the media were measured by ELISA according to the manufacturer’s protocol (RD Systems). Culture media was diluted 1:50 for the Ccl2 assay and 1:3 for the Ccl5 assay.

### Growth inhibition and clonogenic assays

For growth inhibition assays, cells were plated in duplicate 96-well culture dishes at 3 x 10^3^ cells per well. The following day, one plate was treated with RTA 408 and the other was immediately processed for the sulforhodamine B (SRB) assay (time 0) as described in [33]. Cells in the RTA 408-treated plate were processed for the SRB assay 72 hours after the start of treatment. Percentage of growth relative to vehicle-treated cells was calculated using: [(T_i_-T_z_)/(C-T_z_)] x 100 where (T_z_) is the absorbance value at time zero, (C) is absorbance value from vehicle treated wells after 72 hours, and (T_i_) is the absorbance value from wells treated with the drug. Dose-response curves were plotted in GraphPad Prism and GI_50_ values were calculated. For cell counting experiments, MEFs were plated in 6-well culture dishes at 5 x 10^4^ cells per well and treated with RTA 408 the following day. Following treatment, cells were counted using a Vi-CELL XR cell analyzer (Beckman Coulter). For clonogenic assays, wild-type (1 x 10^3^ cells per well) and *Keap1* ^-/-^ (0.5 x 10^3^ cells per well) MEFs were seeded in 6-well dishes. Six hours later, MEFs were treated with RTA 408. After seven days, colonies were fixed with a 1:7 solution of acetic acid:MeOH and stained with 0.5% crystal violet in MeOH. Colonies consisting of ≥50 cells were counted.

### Caspase activity

Caspase activity was determined as described previously [34] using DEVD-AFC (EMD Biosciences) as the substrate.

### NF-κB signaling

For NF-κB-luciferase reporter assays, HeLa NF-κB-Luc (1.9 x 10^4^ cells per well) and A549/NF-κB-Luc (1.6 x 10^4^ cells per well) cells were seeded in 96-well black plates with clear bottoms. Twenty four hours later, cells were pre-treated with DMSO or several concentrations of RTA 408 for one hour and then treated with 10 ng/ml human TNFα for five additional hours. Firefly luciferase activity was measured using the One-Glo Luciferase Assay (Promega, Madison, WI) according to the manufacturer’s instructions. For IκBα western blots, HeLa cells were seeded in a 24-well culture dish at a density of 1 x 10^5^ cells per well. The following day, cells were pre-treated with DMSO or several concentrations of RTA 408 or bardoxolone methyl for six hours. Cells were then treated with 20 ng/ml of human TNFα for five minutes. Cells were immediately lysed in Tricine sample buffer with 2% BME and processed for western blotting as described above.

### Statistical analyses

Statistical significance was determined by repeated measures one-way ANOVA and Dunnett’s multiple comparison test.

## Results

### RTA 408 increases expression of Nrf2 target genes and decreases expression of inflammatory mediators

**Fig 1:**
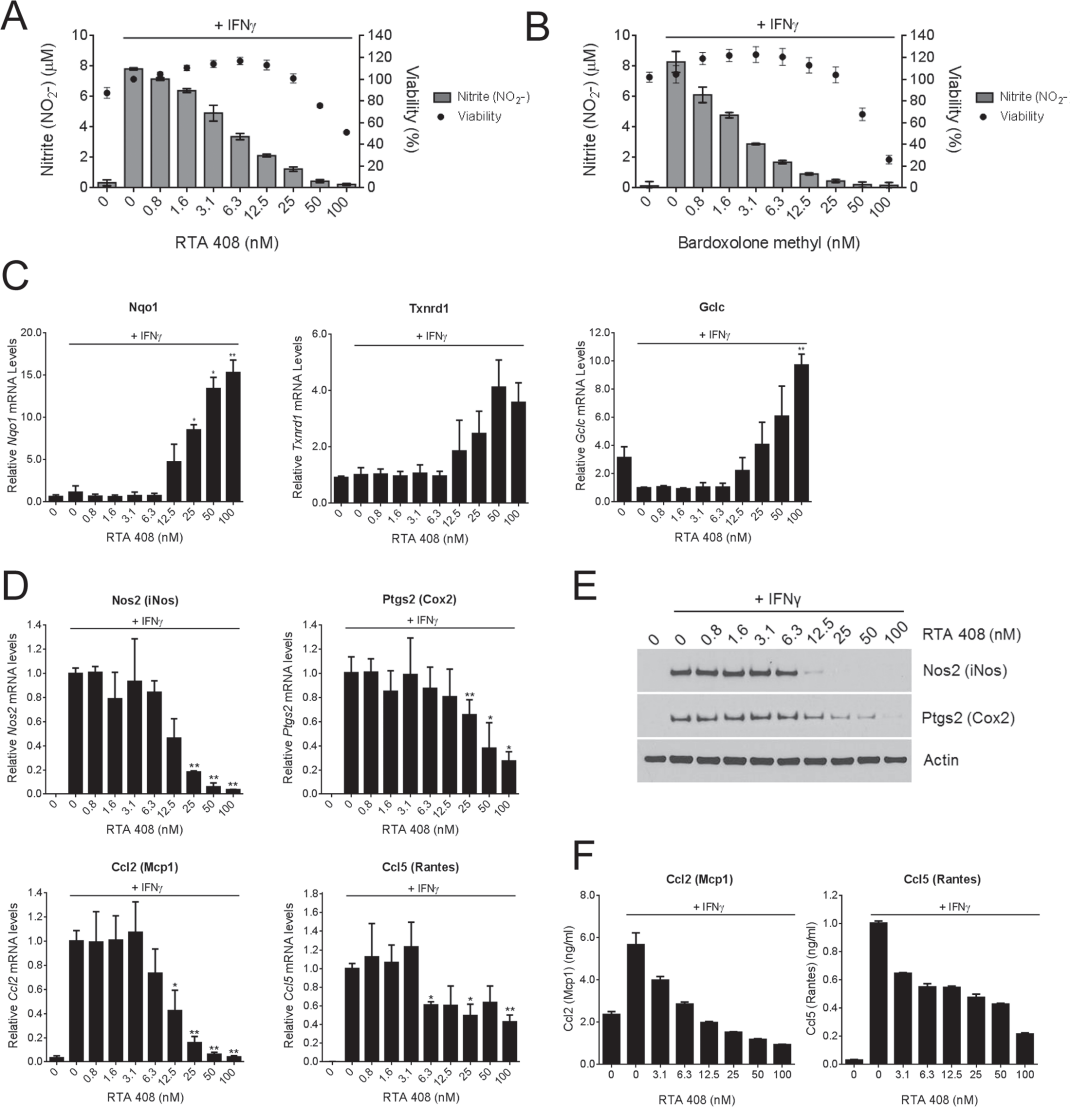
RTA 408 Increases Expression of Nrf2 Target Genes and Decreases Expression of Inflammatory Mediators in RAW 264.7 Cells. RAW 264.7 macrophages were treated with the indicated concentrations of RTA 408 or bardoxolone methyl for two hours, and then treated with 20 ng/mL IFNγ for an additional 24 hours. ***A***, ***B***, Nitrite (NO_2_-) concentrations in the media were measured by Griess reaction and cell viability was assessed using WST-1 reagent. Viability is presented as percent survival relative to survival in cells treated with IFNγ alone. Data are representative of three experiments. ***C***, mRNA levels of Nrf2 target genes *Nqo1*, *Gclc*, and *Txnrd1* were measured by qRT-PCR. Data are presented relative to expression in cells treated with IFNγ alone. Data points are the mean and SD of three experiments. ***D***, mRNA levels of pro-inflammatory genes *Nos2*, *Ptgs2*, *Ccl2*, *and Ccl5* were measured by qRT-PCR. Values are presented relative to expression in cells treated with IFNγ alone. Data points are the mean and SD of three experiments. ***E***, protein levels of Nos2 and Ptgs2 were evaluated by Western blot. Actin served as a loading control. Data are representative of two experiments. ***F***, protein levels of Ccl2 and Ccl5 in the culture media were measured by ELISA. Data are representative of two experiments. For ***C*** and ***D***, statistical significance was determined by repeated measures one-way ANOVA and Dunnett’s multiple comparison test. *, *P* < 0.05; **, *P* <0.01 compared to cells treated with IFNγ alone.

AIMs are potent suppressors of interferon gamma (IFNγ)-induced nitric oxide (NO) production [35], a process central to pro-inflammatory signaling. To evaluate the anti-inflammatory activity of RTA 408, we treated RAW 264.7 mouse macrophage cells with RTA 408 for two hours and then added IFNγ to stimulate NO production and release into the media. RTA 408 dose-dependently reduced NO concentrations in the media (Fig 1A) with an IC_50_ value of 4.4 ± 1.8 nM. The potency of RTA 408 in this assay is similar to that of bardoxolone methyl, which had an IC_50_ value of 1.9 ± 0.8 nM (Fig 1B). Nrf2 activation is required for AIM-mediated NO suppression [3]. Consistent with this, we observed a decrease in nitric oxide synthase 2 (Nos2) protein levels in bardoxolone methyl-treated RAW 264.7 cells, which was attenuated when Nrf2 mRNA levels were reduced by siRNA (S1 Fig). To determine whether RTA 408 activates Nrf2 in IFNγ-treated RAW 264.7 cells, we used qRT-PCR to measure the mRNA levels of three Nrf2 target genes: *Nqo1*, *Txnrd1*, and *Gclc*. IFNγ treatment alone had no effect on *Nqo1* or *Txnrd1* mRNA levels, but reduced basal *Gclc* mRNA levels (Fig 1C). Treatment with RTA 408 dose-dependently increased expression of all three genes, demonstrating that RTA 408 potently activates Nrf2 under inflammatory conditions and can reverse IFNγ-mediated suppression of *Gclc* expression.

AIMs inhibit the expression of pro-inflammatory mediators in an Nrf2-dependent manner [36]. In this study, RTA 408 dose-dependently reduced the mRNA levels of *Nos2* (*iNos*), *Ptgs2* (*Cox2*), *Ccl2* (*Mcp1*), and *Ccl5* (*Rantes*) in IFNγ-treated RAW 264.7 cells (Fig 1D). The reduction in pro-inflammatory gene expression occurred at the same concentrations that increased the levels of Nrf2 target gene expression (≥ 12.5 nM). Treatment with similar concentrations of RTA 408 also reduced the protein levels of pro-inflammatory mediators as measured by Western blot (Fig 1E) or ELISA (Fig 1F). Taken together, these results demonstrate that RTA 408, similar to bardoxolone methyl, potently activates Nrf2 and reduces the levels of several key pro-inflammatory mediators.

### RTA 408 inhibits growth and induces apoptosis in human tumor cell lines

**Fig 2:**
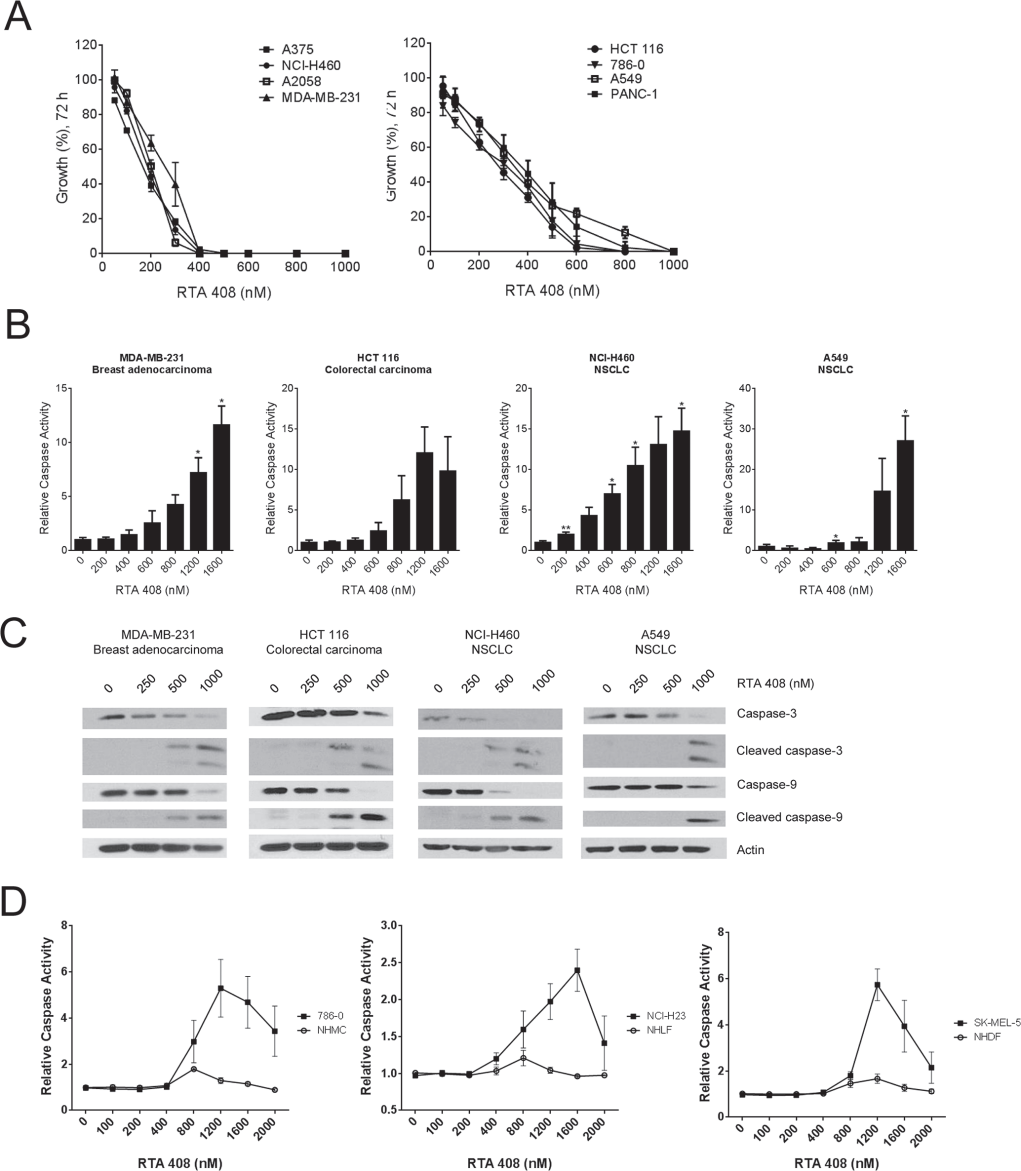
RTA 408 Inhibits Growth and Induces Apoptosis in Human Tumor Cell Lines. ***A***, Cells were treated with RTA 408 for 72 hours and viability was assessed using the SRB assay. Values are presented as percent growth in RTA 408-treated cells relative to growth in vehicle-treated cells. ***B***, Human tumor cell lines were treated with RTA 408 for 24 hours and cleavage of the DEVD-AFC peptide was measured as a surrogate for caspase activity. Data are presented as caspase activity in RTA 408-treated cells relative to activity in vehicle-treated cells. Data points are the mean and SD of three experiments. Statistical significance was determined by repeated measures one-way ANOVA and Dunnett’s multiple comparison test. *, *P* < 0.05; **, *P* < 0.01 compared to vehicle-treated cells. ***C***, Cells were treated with RTA 408 for 24 hours and protein levels of caspase-3 and caspase-9 were evaluated by Western blot. Actin served as a loading control. Data are representative of two experiments. ***D***, Human tumor cell lines and normal primary human cells were treated with RTA 408 for 24 hours and cleavage of the DEVD-AFC peptide was measured as a surrogate for caspase activity. Data are presented as caspase activity in RTA 408-treated cells relative to activity in vehicle-treated cells. Data points are the mean and SD of three experiments.

To evaluate the anticancer activity of RTA 408, we treated a panel of eight human cell lines derived from tumors of different origin with RTA 408 and measured cell growth 72 hours later using the sulforhodamine B (SRB) assay. RTA 408 inhibited the growth of all tumor lines with an average GI_50_ value of 260 ± 74 nM (Fig 2A and Table 1). To determine whether RTA 408 induces apoptosis, we treated the panel of tumor cells with RTA 408 and the caspase substrate, DEVD-AFC, for 24 hours. RTA 408 dose-dependently increased DEVD-AFC cleavage, indicating that RTA 408 treatment triggered caspase activation in cancer cells (Fig 2B and S2 Fig). Caspase-3 and caspase-9 cleavage was also observed by western blot at the same concentrations of RTA 408 that increased DEVD-AFC cleavage (Fig 2C and S2 Fig). We also evaluated the effect of RTA 408 on DEVD-AFC cleavage in pairs of cancer and normal cells derived from lung, skin, and kidney. In all cases, RTA 408 dose-dependently increased caspase activity in the cancer cells, but not in the corresponding normal primary cells (Fig 2D). The decrease in caspase activity in the cancer cells at concentrations of RTA 408 greater than 1200 nM likely reflects fewer cells due to cell death.

**Table 1:** Human Tumor Cell Line Growth Inhibition Following RTA 408 Treatment for 72 hours.

Cell Line	Cancer Type	GI_50_ (nM)^a^
A375	Melanoma	159.1 ± 8.6
NCI-H460	Lung carcinoma (NSCLC)	182.5 ± 19.7
A2058	Melanoma	199.6 ± 8.1
MDA-MB-231	Breast adenocarcinoma	262.6 ± 34.7
HCT 116	Colorectal carcinoma	272.7 ± 27.2
786–0	Renal cell adenocarcinoma	303.6 ± 25.4
A549	Lung carcinoma (NSCLC)	335.3 ± 4.8
PANC-1	Pancreatic epithelioid carcinoma	363.6 ± 53.3

^a^ Values are mean ± standard deviation of three independent experiments.

### RTA 408 does not increase cell proliferation or survival

**Fig 3:**
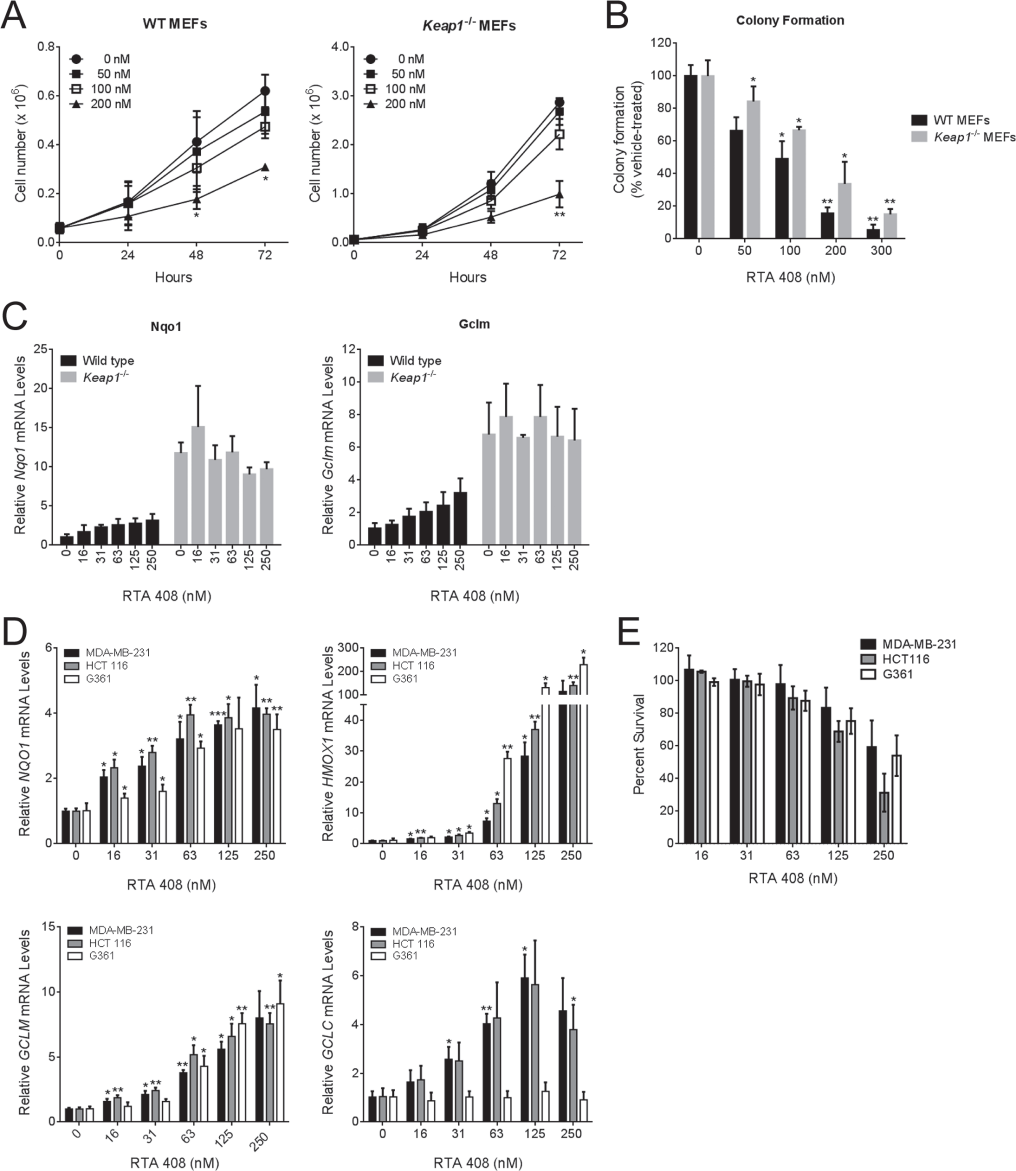
RTA 408 Inhibits Proliferation and Colony Formation in Wild-Type and *Keap1*-/- MEFs. ***A***, Growth of WT and *Keap1* ^-/-^ MEFs treated with RTA 408. MEFs were treated with RTA 408 and cells were counted at 24-hour intervals. Statistical significance was determined by repeated measures one-way ANOVA and Dunnett’s multiple comparison test. *, *P* < 0.05; **, P < 0.01 compared to vehicle-treated cells at the same time point. ***B***, Colony formation by WT and *Keap1* ^-/-^ MEFs treated with RTA 408. Statistical significance was determined by repeated measures one-way ANOVA and Dunnett’s multiple comparison test. *, *P* < 0.05; **, P < 0.01 compared to vehicle-treated cells. ***C***, Nrf2 target gene expression in WT and *Keap1* ^-/-^ MEFs treated with RTA 408 for 18 hours. mRNA levels of *Nqo1* and *Gclm* were measured by qRT-PCR. Values are presented as fold-induction relative to vehicle-treated WT MEFs. ***D***, RTA 408 increases expression of Nrf2 target genes in human tumor cell lines. MDA-MB-231, HCT 116, and G361 cells were treated with the indicated concentrations of RTA 408 for 18 hours. mRNA levels of *NQO1*, *HMOX1*, *GCLM*, and *GCLC*, were measured by qRT-PCR. Data are presented as fold-induction relative to vehicle-treated cells for each cell line. Statistical significance was determined by repeated measures one-way ANOVA and Dunnett’s multiple comparison test.*, *P* < 0.05; **, *P* <0.01; ***, *P* < 0.001 compared to vehicle-treated cells. ***E***, MDA-MB-231, HCT 116, and G361 cells were treated with RTA 408 for 48 hours and cell viability was determined using the SRB assay. Data are presented as percent survival relative to survival in vehicle-treated cells. In all panels, data points are the mean of three independent experiments and error bars are SD.

*KEAP1* inactivating and *NRF2* activating mutations have been identified in human tumor biopsies [37]. The observation that constitutive Nrf2 activation is correlated with poor patient survival and increased therapeutic resistance has raised the question of whether pharmacological activation of Nrf2 could increase cell proliferation or survival. Loss of Keap1 in murine embryonic fibroblasts (MEFs) results in constitutive Nrf2 activation and increased MEF survival [30,38]. In contrast to these findings, RTA 408 dose-dependently decreased growth (Fig 3A) and inhibited colony formation (Fig 3B) in WT and *Keap1* ^*-/-*^ MEFs. At growth-inhibiting concentrations, RTA 408 increased *Nqo1* and *Gclm* mRNA levels in WT MEFs, but not in *Keap1* ^-/-^ MEFS, which exhibited elevated basal levels of these Nrf2 target genes (Fig 3C). To extend these findings to human tumor cells, we selected three cell lines and assessed whether RTA 408 could inhibit tumor cell growth at doses that activated Nrf2. RTA 408 increased expression of Nrf2-target genes in all three cell lines (Fig 3D). We did not observe an increase in *GCLC* mRNA levels in the G-361 melanoma cell line following RTA 408 treatment; the molecular mechanism underlying this is not known. Despite robust activation of Nrf2, RTA 408 dose-dependently decreased survival of all three cells lines at doses ≤ 250 nM (Fig 3E). We obtained similar results when we treated the cells with equivalent concentrations of bardoxolone methyl (S3 Fig). These data demonstrate that RTA 408 activates Nrf2 in a Keap1-dependent manner; however, pharmacologic Nrf2 activation by RTA408 does not correlate with increased cell proliferation or cell survival.

### RTA 408 inhibits NF-κB and activates JNK in tumor cells

**Fig 4:**
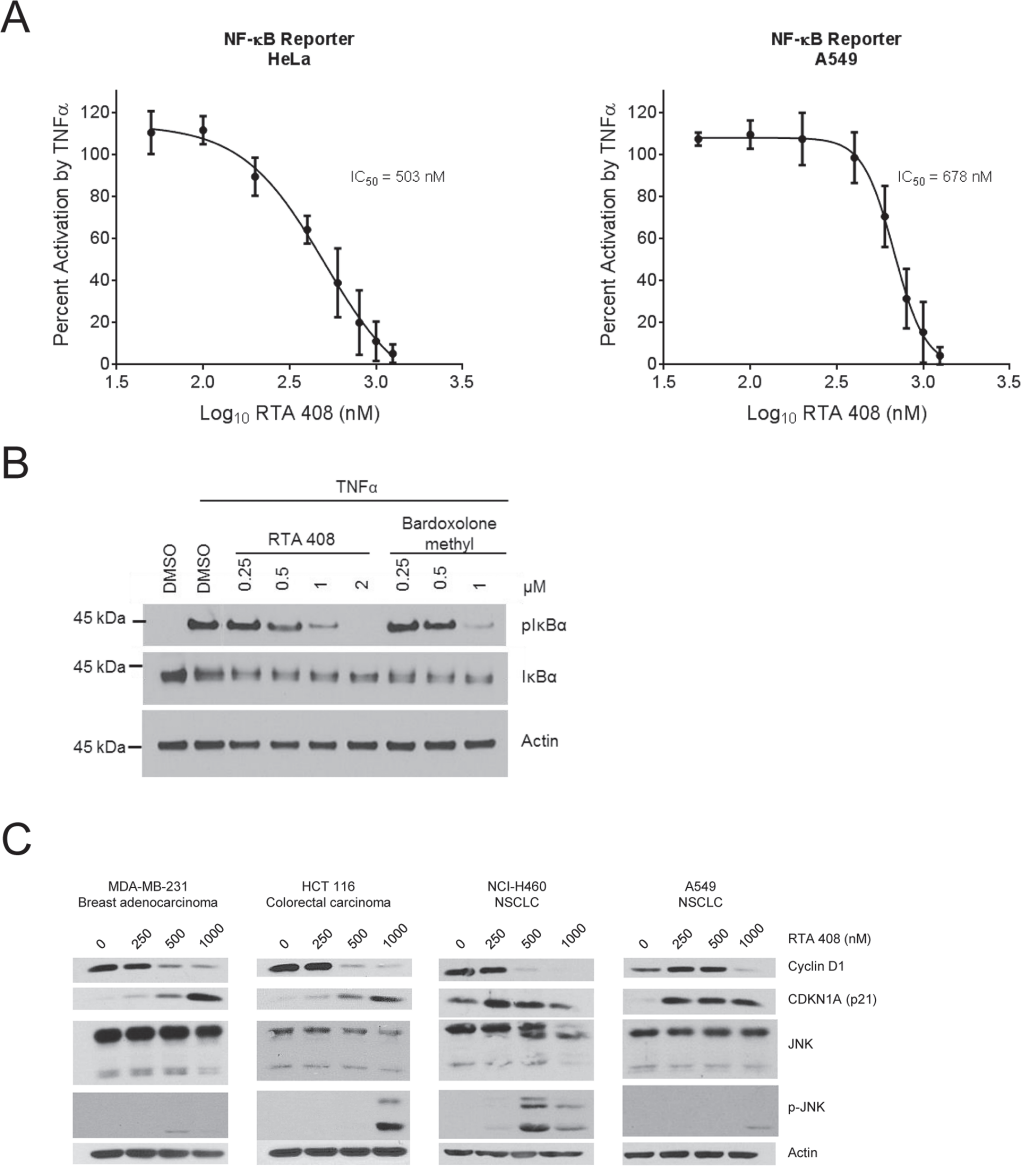
RTA 408 Inhibits NF-κB and Activates JNK. ***A***, HeLa/NF-κB-Luc or A549/NF-κB-Luc cells were treated with RTA 408 for one hour and then treated with 10 ng/mL TNFα. Five hours later, luminescence was measured to assess NF-κB activity. Data are presented as percent activity relative to cells treated with TNFα alone. Data points for HeLa and A549 are the mean and SD of three and four experiments, respectively. ***B***, HeLa cells were pre-treated with RTA 408 or bardoxolone methyl for 6 hours at the indicated concentrations followed by a five-minute treatment with TNFα. Protein levels of phospho-IκBα and total IκBα were evaluated by western blot. Actin was used as loading control. Data are representative of four experiments. ***C***, Cells were treated with RTA 408 for 24 hours and protein levels of cyclin D1, CDKN1A (p21), and total and phospho-JNK were evaluated by western blot. Actin served as a loading control. Data are representative of two experiments.

AIMs inhibit tumor cell growth and induce apoptosis by modulating several key pathways that regulate survival [9,10]. AIMs have been shown to directly bind to cysteine 179 in IKKβ and inhibit NF-κB signaling [11–13]. To assess whether RTA 408 inhibits NF-κB signaling, we used HeLa and A549 cells that were stably transfected with NF-κB-luciferase reporter constructs. When we pre-treated the cells with RTA 408 for one hour, and then stimulated with TNFα for five hours, we observed a dose-dependent decrease in NF-κB-dependent luciferase activity in both cell lines (Fig 4A). RTA 408 also inhibited TNFα-induced phosphorylation of IκBα in HeLa cells at concentrations similar to those of bardoxolone methyl (Fig 4B), consistent with an inhibitory effect on IKKβ. AIMs have also been shown to decrease cyclin D1 (an NF-κB target gene) and increase CDKN1A (p21) levels in cultured breast cancer cells [39,40]. Furthermore, bardoxolone methyl reduced cyclin D1 levels in tumors from xenograft and transgenic mouse models [17,41], and in tumor biopsies from patients enrolled in a phase 1 clinical trial (ClinicalTrials.gov ID: NCT00529438) [27]. In the present study, RTA 408 reduced cyclin D1 and increased CDKN1A protein levels in all eight tumor lines (Fig 4C and S4 Fig). JNK has also been shown to be phosphorylated in response to treatment with AIMs and is required for induction of AIM-mediated apoptosis in cancer cells [14,42]. In the present study, we observed JNK phosphorylation in seven of the eight cancer cell lines upon treatment with 500 or 1000 nM RTA 408 (Fig 4C and S4 Fig). Phosphorylation of JNK in response to RTA 408 occurred at concentrations that increased cleavage of caspase-3 and caspase-9 (Fig 2C and S2 Fig). These results suggest that RTA 408 directly inhibits tumor cell growth and activates apoptosis in a manner similar to bardoxolone methyl and other AIMs.

## Discussion

The results of the present study demonstrate for the first time that RTA 408 is a potent anticancer agent with an activity profile similar to that of bardoxolone methyl. The AIMs were originally developed as chemopreventive agents and were initially optimized for their ability to suppress the production of NO in mouse macrophages [43]. Several years after their discovery, Dinkova-Kostova et al. [3] observed a positive correlation between the potency of these compounds in NO suppression and Nrf2 activation assays, suggesting that the two activities were mechanistically linked. Additional studies expanded on these findings and demonstrated that Nrf2 silencing attenuates AIM-mediated suppression of key pro-inflammatory cytokines [36]. Consistent with this, we found that low concentrations of RTA 408 (≤ 25 nM) suppressed IFNγ-induced NO production and pro-inflammatory cytokine expression and increased Nrf2 target gene expression in the RAW 264.7 macrophage cell line. These results demonstrate that, similar to bardoxolone methyl, RTA 408 has potent antioxidant and anti-inflammatory activity.

Nrf2 activation counteracts chemical carcinogens by increasing the levels of antioxidant, detoxification, and conjugation enzymes [44]. The concerted action of these enzymes reduces macromolecular damage and prevents genomic mutation by decreasing reactive oxygen species and promoting export of the toxin. However, the anticancer activity of Nrf2 is not limited to its defense against chemical carcinogens; Nrf2 also suppresses invasion and metastasis of established tumor cells [45–47]. *Nrf2* ^-/-^ mice develop more metastatic lung nodules than wild-type mice and the nodules have higher levels of inflammatory cell infiltration [47]. Selective deletion of Nrf2 in the myeloid lineage also increases lung metastases, highlighting the important role that Nrf2 plays in the metastatic niche [24]. In tumor-associated myeloid-derived suppressor cells (MDSCs), loss of Nrf2 exacerbates the high level of reactive oxygen species (ROS) [24], which promote tumor-mediated immune suppression by inhibiting innate and adaptive immunity [48]. Conversely, Nrf2 activation by AIMs reduces ROS levels in MDSCs, reverses tumor-mediated suppression of CD8^+^ T cell activity and proliferation, and inhibits lung metastasis [24,25]. AIMs also alter the cancer-promoting activity of other cells found within the tumor microenvironment, including dendritic cells [23], bone marrow stromal cells [49], and the vascular endothelia [22]. In transgenic models of cancer, AIMs delay tumor development and increase survival [40,41,50]. Therefore as a potent activator of Nrf2, RTA 408 has the potential to inhibit tumor growth and metastasis by reducing both ROS and inflammation within the tumor microenvironment and the metastatic niche.

Nrf2 is constitutively active in some human tumors, and has been associated with drug resistance and poor prognosis [37]. Accordingly, there is general concern that the use of pharmacological Nrf2 activators in the clinic may promote tumor growth. Despite activation of Nrf2 by AIMs, preclinical studies have consistently demonstrated the anticancer activity of these compounds [51]. Consistent with this, RTA 408 did not promote cancer cell growth or viability at concentrations that increased Nrf2 activity (Fig 3). Several factors could contribute to the different effects of constitutive vs. pharmacological Nrf2 activation on cancer cell growth, including the magnitude and duration of Nrf2 target gene expression [52]; the levels of other Keap1-interacting proteins, such as IKKβ and BCL2 [53–55]; and AIM-mediated activation or inhibition of other pathways that affect cancer cell survival, such as IKKβ (NF-κB), STAT3, JNK, Cyclin D1, CDKN1A (p21) [9,10].

**Fig 5:**
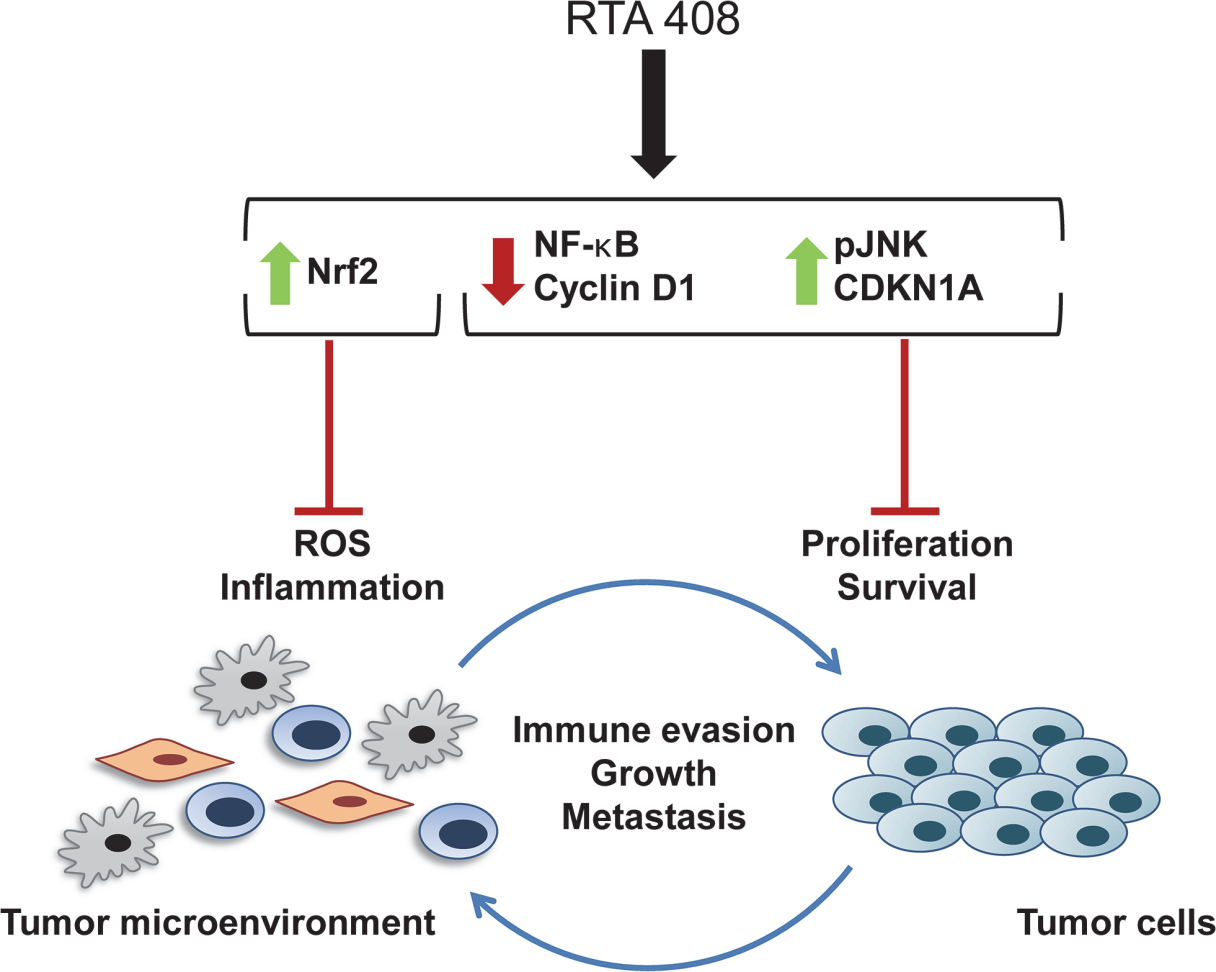
RTA 408 Mechanism of Action. The coordinated anticancer activities of RTA 408 affect both tumor cells and cells within the tumor microenvironment. By activating Nrf2 and reducing ROS and inflammation in the tumor microenvironment, RTA 408 reverses tumor-mediated immune suppression and prevents tumor growth and metastasis. Within the tumor cells, transient Nrf2 activation by RTA 408 does not promote growth or survival. RTA 408 also modulates the activity of oncogenic signaling pathways (such as NF-κB, cyclin D1, JNK, and CDKN1A (p21)) and promotes growth arrest and apoptosis of tumor cells.

In summary, RTA 408 is a novel AIM with potent antioxidant, anti-inflammatory, and anticancer activity. The multifaceted anticancer mechanism of action of AIMs is summarized in Fig 5. By reducing oxidative stress and inflammation in the tumor microenvironment, AIMs attenuate the immunosuppressive activity of tumor-associated MDSCs, inhibit angiogenesis, and prevent metastasis [22,24,25,47]. By modulating several key oncogenic signaling pathways, including NF-κB, AIMs directly inhibit growth and induce apoptosis in tumor cells. The data herein demonstrate that the coordinated anticancer activities of RTA 408 are similar to those of bardoxolone methyl, which was well-tolerated at doses that demonstrated target engagement in patients with advanced malignancies [27]. The safety of RTA 408 is currently being evaluated in patients with metastatic non-small cell lung cancer or melanoma (clinicaltrials.gov ID: NCT02029729).

## Supporting Information

S1 Fig Bardoxolone Methyl Reduces Nos2 Levels in an Nrf2-Dependent Manner in RAW 264.7 Macrophages. (TIF)

S2 Fig RTA 408 Induces Apoptosis in Human Tumor Lines. (TIF)

S3 Fig Bardoxolone Methyl Activates Nrf2 and Inhibits Proliferation in Human Tumor Lines. (TIF)

S4 Fig RTA 408 Reduces Cyclin D1 Levels and Increases CDKN1A (p21) and p-JNK Levels in Human Tumor Lines. (TIF)

S1 Table PCR primer information. (DOCX)

S2 Table Antibody information. (DOCX)
