# Supplementary figures and images for: RTA 408, A Novel Synthetic Triterpenoid with Broad Anticancer and Anti-Inflammatory Activity
Source: PLoS One. 2015 Apr 21;10(4):e0122942. doi: 10.1371/journal.pone.0122942 (PMC4405374; doi:10.1371/journal.pone.0122942)

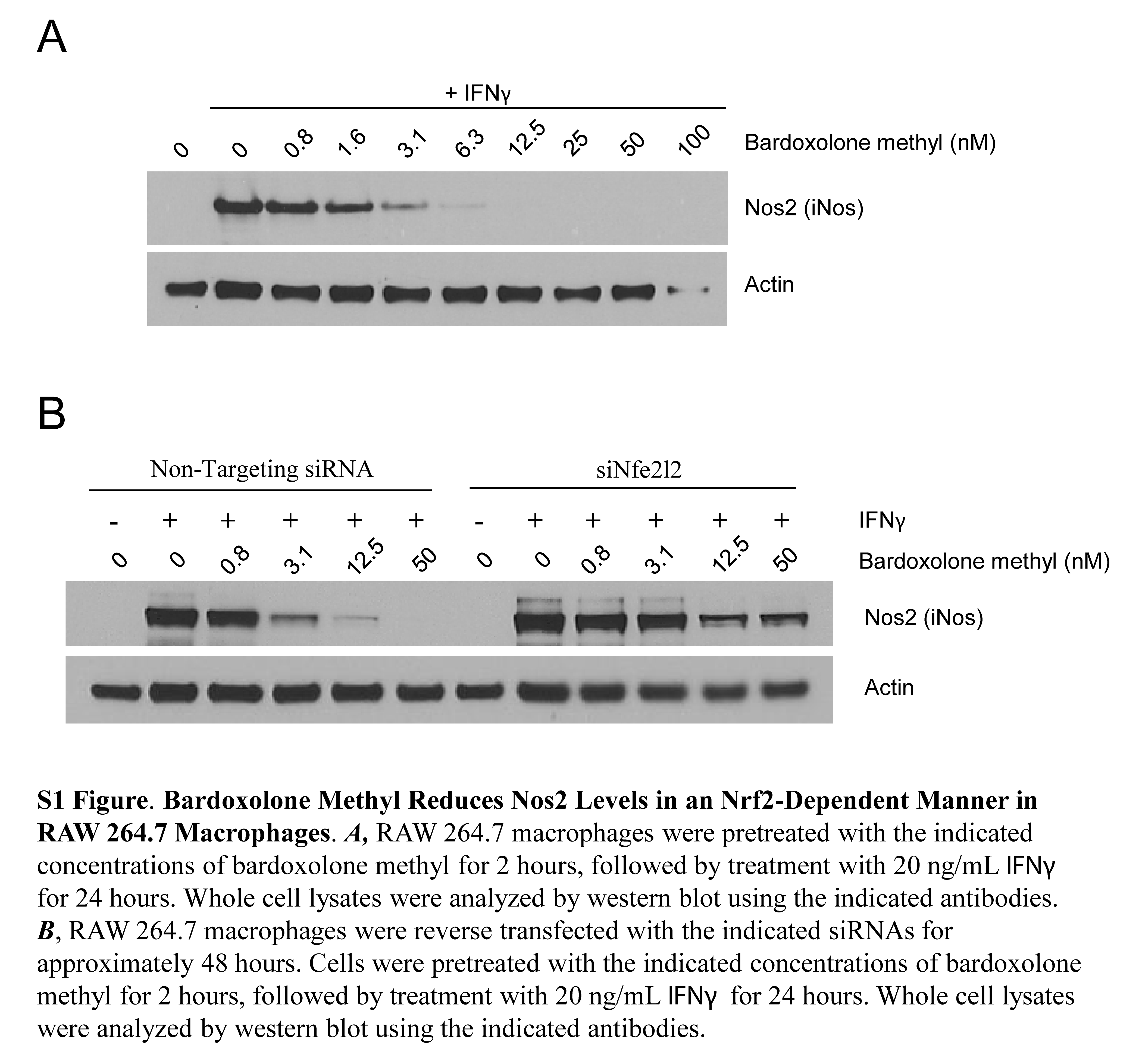

Supplement: S1 Fig — (TIF) [file pone.0122942.s001.tif]

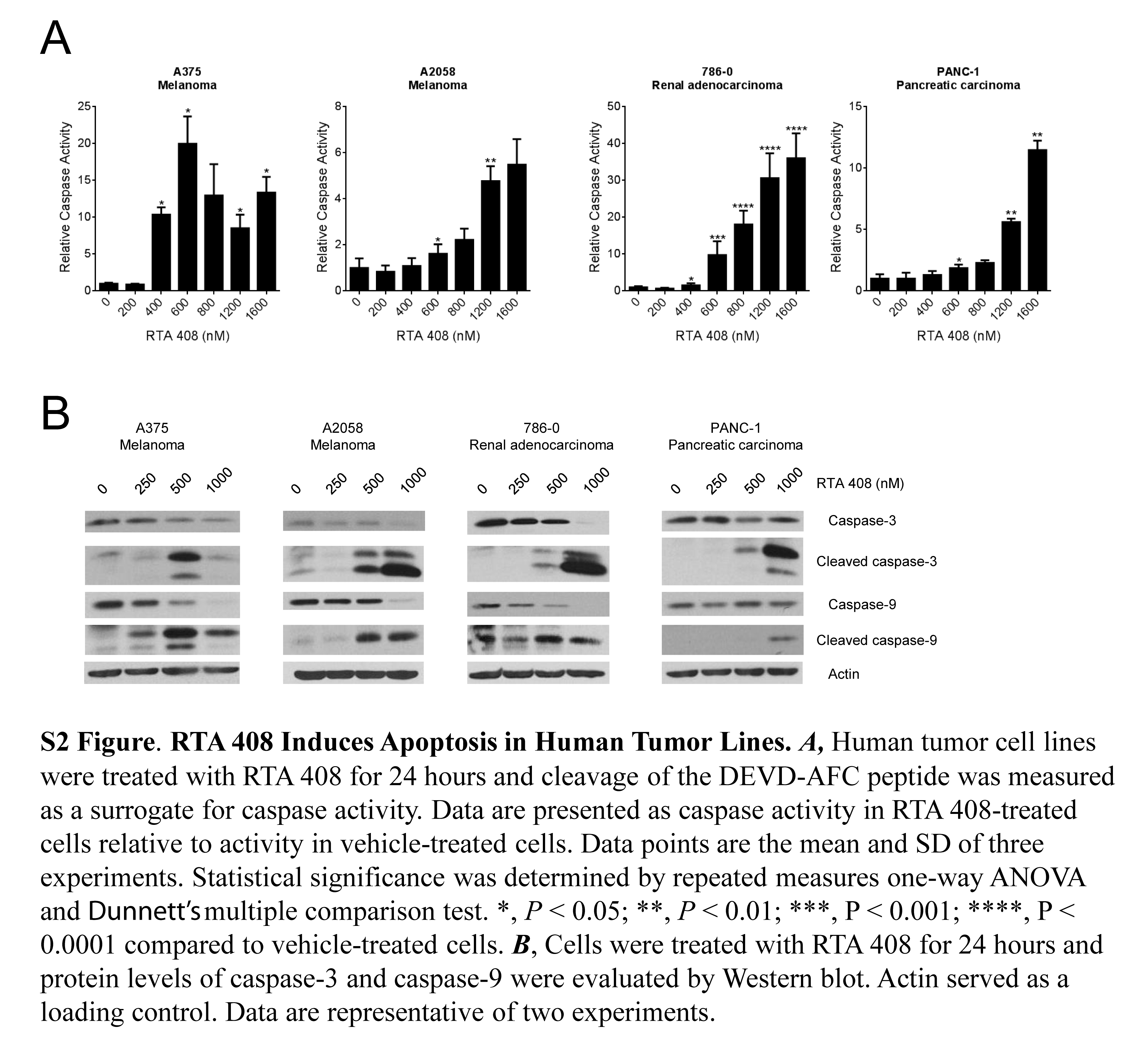

Supplement: S2 Fig — (TIF) [file pone.0122942.s002.tif]

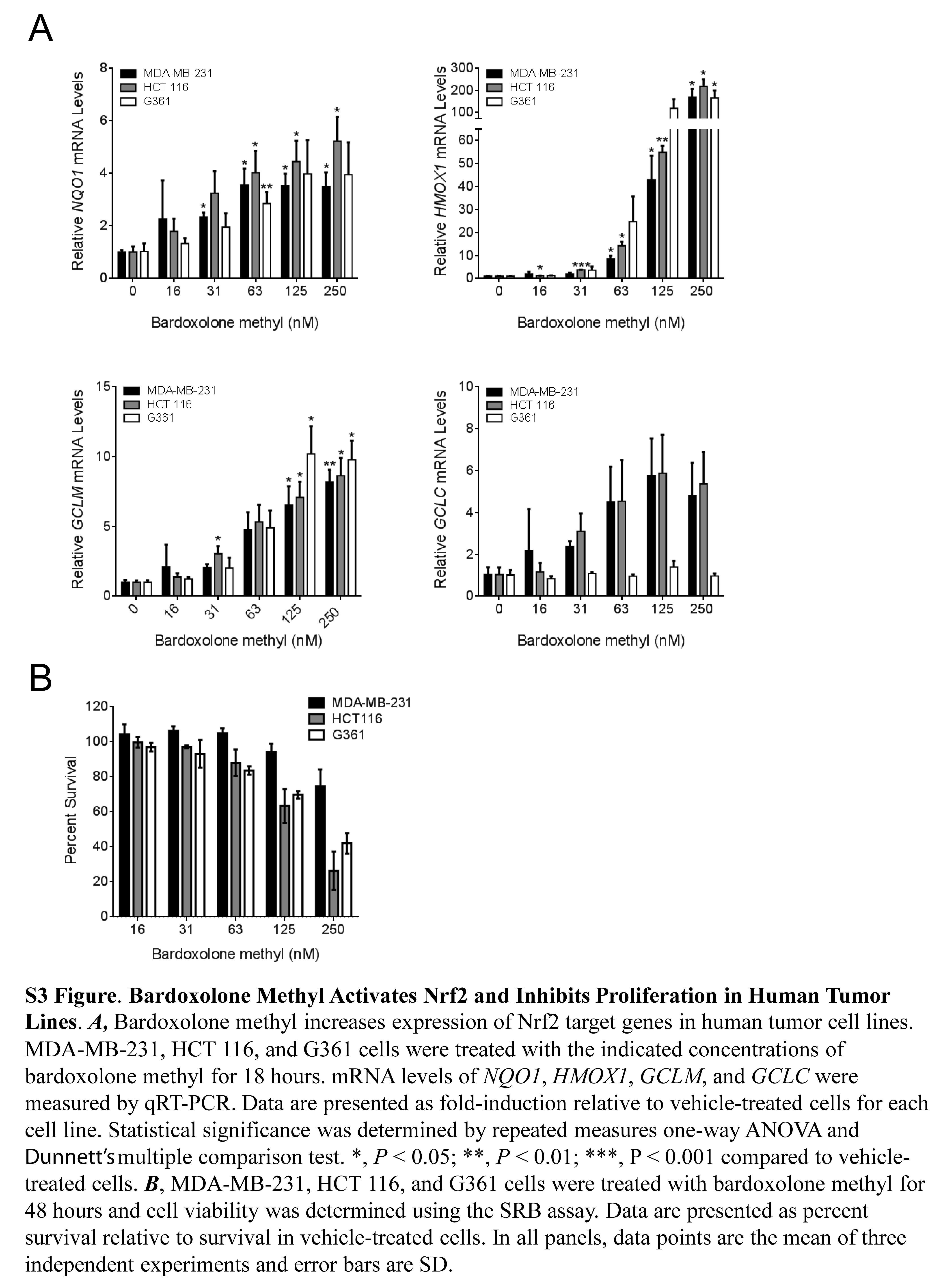

Supplement: S3 Fig — (TIF) [file pone.0122942.s003.tif]

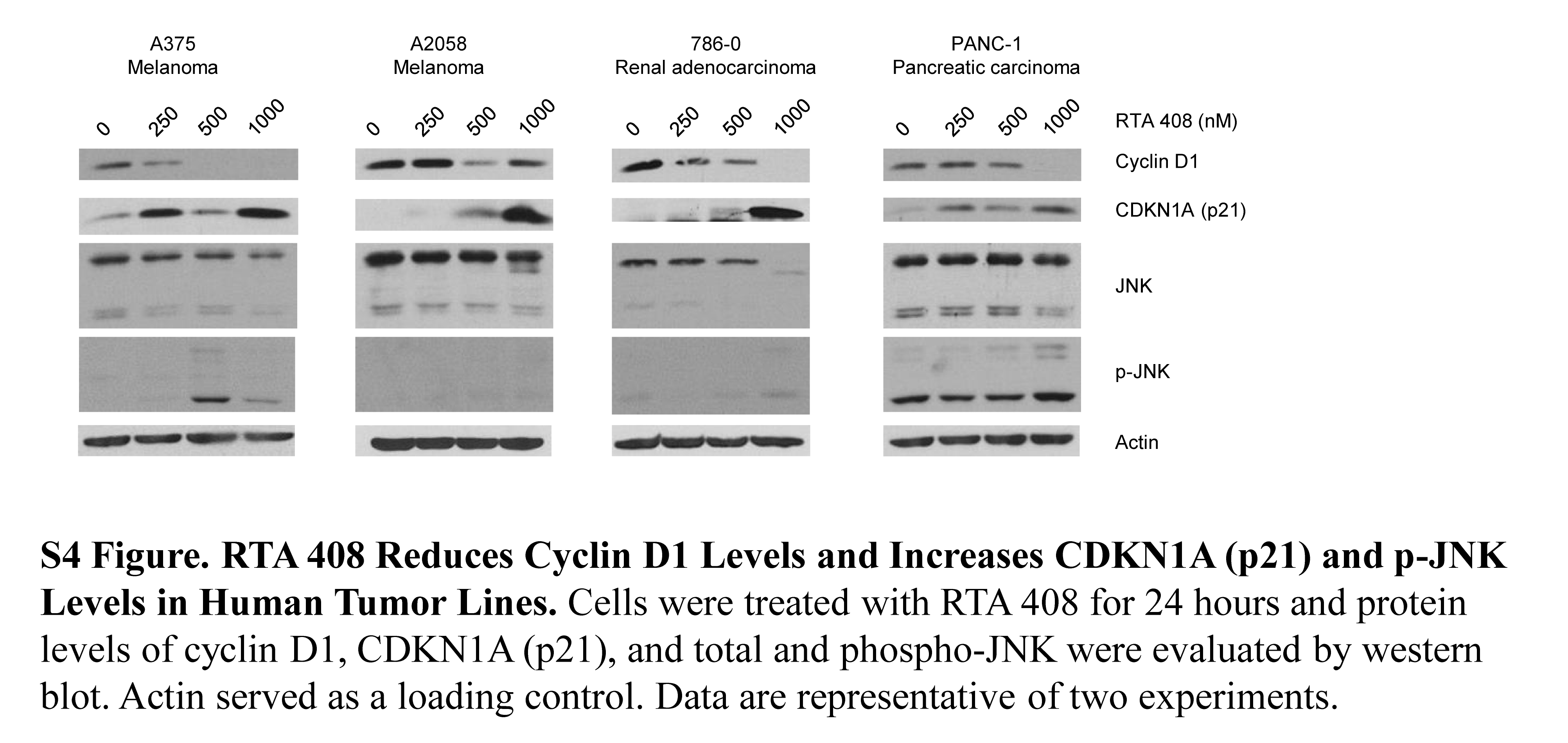

Supplement: S4 Fig — (TIF) [file pone.0122942.s004.tif]
