# Supplementary material for: RTA 408, A Novel Synthetic Triterpenoid with Broad Anticancer and Anti-Inflammatory Activity
Source: PLoS One. 2015 Apr 21;10(4):e0122942. doi: 10.1371/journal.pone.0122942 (PMC4405374; doi:10.1371/journal.pone.0122942)
Supplement: S1 Table — (DOCX) [file pone.0122942.s005.docx]

**S1 Table. PCR primer information**

| **Species** | **Gene Symbol** | **Forward primer** | **Reverse primer** |
| --- | --- | --- | --- |
| Human | RPS9 | 5’-GATGAGAAGGACCCCACGGCGTCTG-3’ | 5’-GAGACAATCCAGCAGCCCAGGAGGG-3’ |
| Human | NQO1 | 5’-AAAACACTGCCCTCTTGTGG-3’ | 5’-GTGCCAGTCAGCATCTGGTA-3’ |
| Human | GCLM | 5’-GCTGTGGCTACTGCGGTATT-3’ | 5’-ATCTGCCTCAATGACACCAT-3’ |
| Human | HMOX1 | 5’-TCCGATGGGTCCTTACACTC-3’ | 5’-TAGGCTCCTTCCTCCTTTCC-3’ |
| Human | GCLC | 5’-CTTGTAGTCAGGATGGTTTGCG-3’ | 5’-TCCTGGACTGATCCCAATTCTG-3’ |
| Mouse | Ccl2 | 5’-ATGCAGTTAATGCCCCACTC-3’ | 5’-TTCCTTATTGGGGTCAGCAC-3’ |
| Mouse | Ccl5 | 5’-GTGCCCACGTGAAGGAGTAT-3’ | 5’-ATCCCCAGCTGGTTAGGACT-3’ |
| Mouse | Gclc | 5’-CAATGGGAAGGAAGGGGTAT-3’ | 5’-TCAGGATGGTTTGCAATGAA-3’ |
| Mouse | Nos2 | 5’-ATTGCTCCCTTCCGAAGTTT-3’ | 5’-TGCAGGATGTCCTGAACGTA-3’ |
| Mouse | Nqo1 | 5’-TCGGGCTAGTCCCAGTTAGA-3’ | 5’-AAAGAGCTGGAGAGCCAACC-3’ |
| Mouse | Ptgs2 | 5’-TAGGCTGTTGGAATTTACGC-3’ | 5’-TCATTTCTTGATGCCCGAAT-3’ |
| Mouse | Rpl19 | 5’-TCAGGCTACAGAAGAGGCTTGC-3’ | 5’-ACAGTCACAGGCTTGCGGATG-3’ |
