# Supplementary material for: RTA 408, A Novel Synthetic Triterpenoid with Broad Anticancer and Anti-Inflammatory Activity
Source: PLoS One. 2015 Apr 21;10(4):e0122942. doi: 10.1371/journal.pone.0122942 (PMC4405374; doi:10.1371/journal.pone.0122942)
Supplement: S2 Table — (DOCX) [file pone.0122942.s006.docx]

**S2 Table. Antibody Information**

| **Antibody Name** | **Type** | **Host species** | **Dilution** | **Commercial Supplier** | **Catalog Number** |
| --- | --- | --- | --- | --- | --- |
| Actin, clone C4 | Monoclonal | Mouse | 1:40,000 | Millipore | MAB1501 |
| Caspase-3 (8G10) | Monoclonal | Rabbit | 1:2000 | Cell Signaling Technology | 2870 |
| CDKN1A (p21) (N-20) | Polyclonal | Rabbit | 1:1000 | Santa Cruz Biotechnology | sc-469 |
| Cleaved Caspase-3 (Asp175) | Polyclonal | Rabbit | 1:1000 | Cell Signaling Technology | 9665 |
| Caspase-9 | Polyclonal | Rabbit | 1:1000 | Cell Signaling Technology | 9502 |
| Cyclin D1 (DCS-6) | Monoclonal | Mouse | 1:1000 | Santa Cruz Biotechnology | sc-20044 |
| IκBα | Polyclonal | Rabbit | 1:500 | Santa Cruz Biotechnology | sc-371 |
| p-IκBα | Monoclonal | Mouse | 1:500 | Cell Signaling Technology | 9246 |
| JNK | Polyclonal | Rabbit | 1:5000 | Cell Signaling Technology | 9252 |
| p-JNK | Monoclonal | Mouse | 1:1000 | Cell Signaling Technology | 9255 |
| Nos2 (M-19) | Polyclonal | Rabbit | 1:200 | Santa Cruz Biotechnology | sc-650 |
| Ptgs2 (Cox-2) (M-19) | Polyclonal | Goat | 1:500 | Santa Cruz Biotechnology | sc-1747 |
